# Mass drug administration of azithromycin for trachoma reduces the prevalence of genital *Chlamydia trachomatis* infection in the Solomon Islands

**DOI:** 10.1136/sextrans-2015-052439

**Published:** 2016-02-17

**Authors:** M Marks, C Bottomley, H Tome, R Pitakaka, R Butcher, O Sokana, H Kako, A W Solomon, D C Mabey

**Affiliations:** 1 Faculty of Infectious and Tropical Diseases, Clinical Research Department, London School of Hygiene & Tropical Medicine, London, UK; 2 Hospital for Tropical Diseases, University College London Hospitals NHS Trust, London, UK; 3 Department of Infectious Diseases Epidemiology, London School of Hygiene & Tropical Medicine, London, UK; 4 Nursing Division, Honiara City Council, Honiara, Solomon Islands; 5 National Referral Hospital, Honiara, Solomon Islands; 6 Eye Health Department, Ministry of Health and Medical Services, Honiara, Solomon Islands; 7 Department of STI and HIV Prevention, Ministry of Health and Medical Services, Honiara, Solomon Islands

**Keywords:** AZITHROMYCIN, CHLAMYDIA TRACHOMATIS, NEISSERIA GONORRHOEA

## Abstract

**Objectives:**

*Chlamydia trachomatis* is the most common bacterial sexually transmitted infection and is frequently asymptomatic; ocular *C. trachomatis* strains cause trachoma. Mass drug administration (MDA) of azithromycin for trachoma might also reduce the prevalence of genital *C. trachomatis*. In a survey conducted in the Solomon Islands in 2014, prior to MDA, the prevalence of genital *C. trachomatis* was 20.3% (95% CI 15.9% to 25.4%). We conducted a survey to establish the impact of MDA with azithromycin on genital *C. trachomatis*.

**Methods:**

Women attending three community outpatient clinics, predominantly for antenatal care, 10 months after MDA with azithromycin given for trachoma elimination, were enrolled in this survey. Self-taken high vaginal swabs were for *C. trachomatis* and *Neisseria gonorrhoeae* using the BD Probetec strand displacement assay.

**Results:**

298 women were enrolled. *C. trachomatis* infection was diagnosed in 43 women (14.4%, 95% CI 10.6% to 18.9%) and *N. gonorrhoeae* in 9 (3%, 95% CI 1.4% to 5.7%). The age-adjusted OR for *C. trachomatis* infection was consistent with a significant decrease in the prevalence of *C. trachomatis* following MDA (OR 0.58, 95% CI 0.37 to 0.94, p=0.027). There was no change in the prevalence of *N. gonorrhoeae* between following MDA (OR 0.51, 95% CI 0.22 to 1.22, p=0.13).

**Conclusions:**

This study demonstrated a 40% reduction in the age-adjusted prevalence of genital *C. trachomatis* infection following azithromycin MDA for trachoma elimination.

## Background

Sexually transmitted infections (STIs) remain a major public health problem and their prevalence is particularly high in resource-limited settings. Worldwide, *Chlamydia trachomatis* and *Neisseria gonorrhoeae* are the most common bacterial STIs;^1^ they may lead to infertility, adverse pregnancy outcomes and an increased risk of HIV transmission.^2–4^ Infection with *C. trachomatis* is frequently asymptomatic. In some high-income countries, routine screening of high-risk groups is encouraged in order to detect *C. trachomatis* infection, but screening is not available where resources are more limited. Mass treatment has been advocated for STI control in high-risk groups in low-income and middle-income countries,^5^ but few studies have evaluated its long-term impact.

The prevalence of bacterial STIs is extremely high in the Western Pacific Region.^6–9^ In clinic based studies conducted in Papua New Guinea, the Solomon Islands and New Caledonia, the prevalence of *C. trachomatis* was estimated to be 11.1%, 20% and 9%, respectively, and the prevalence of *N. gonorrhoeae* was 9.7%, 5.1% and 3.5%, respectively. In a community based survey in Samoa, the prevalence of *C. trachomatis* was 36%.^8^

These studies demonstrate that STIs are a major public health challenge in the Pacific region; innovative strategies are needed to tackle this epidemic.^10^ Some authors have suggested that, given the extremely high prevalence of infection, population-based mass treatment with azithromycin should be considered as a strategy,^8^ but there are limited data to support such an approach.

The Solomon Islands is an archipelago of approximately 1000 islands, located between Papua New Guinea and Vanuatu. It has a population of approximately 500 000.^11^ In a study conducted in 2014, we found the prevalence of genital *C. trachomatis* to be 20% among women attending clinics in the capital and this rose to 30% in women aged under 30 years. The Solomon Islands are also endemic for trachoma, a blinding eye disease caused by infection with ocular strains of *C. trachomatis.*^12^ Mass drug administration (MDA) to the entire population, irrespective of age or gender, of oral azithromycin (20 mg/kg, to a maximum of 1 g in adults) forms the cornerstone of the WHO SAFE strategy for the elimination of trachoma^13^ and might be anticipated to also clear genital *C. trachomatis* infections. The Solomon Islands recently undertook countrywide azithromycin MDA for the purposes of trachoma control, completing the first round in Honiara in September 2014. Following the same methods in the same locations as we used in a pre-MDA survey of STI prevalence,^6^ we conducted a repeat survey of women attending outpatient clinics in Honiara, Solomon Islands approximately 10 months following the MDA to explore any collateral impact on genital infection with *C. trachomatis*.

## Methodology

Primary healthcare to the population of Honiara is generally provided by nurse-led clinics that offer a full range of outpatient services, including general medicine, antenatal care and screening for STIs. All services are free at the point of delivery.

### Study population

We used the same methodology for the survey as in our pre-MDA survey.^6^ All women aged 16–49 years attending three public outpatient clinics in Honiara during a 5-day period in July 2015 were invited to participate in the present survey. Clinic attendees were predominantly presenting for antenatal care. Nursing staff fluent in local dialects explained the study and obtained written informed consent. We did not collect detailed information on the three individuals who declined to participate in the study.

With women who gave written, informed consent to participate, study staff completed a brief questionnaire, including information on the participant's age, marital status, previous treatment for STIs, the presence or absence of current symptoms consistent with an STI and whether the participant had received azithromycin as part of the Ministry of Health and Medical Services’ trachoma elimination campaign. Women were then asked to self-collect two high-vaginal swab samples. Both swabs were collected according to kit manufacturer's guidelines. The first swab (intended for primary diagnostic purposes) was transferred to the National Referral Hospital (NRH) on the day of collection. The second swab was frozen in a dry cryotube on the same day for future analysis. As the purpose of the study was to examine the impact of azithromycin MDA on genital *C. trachomatis*, samples for HIV or syphilis were not collected unless these were indicated, according to local protocols, as part of the patient's routine clinic care.

### Laboratory testing

First swabs were tested within 24 h of collection at the NRH, using the BD Probetec strand displacement amplification assay (Beckton Dickinson, Franklin Lakes, New Jersey, USA) for the simultaneous detection of *C. trachomatis* and *N. gonorrhoeae* DNA, according to manufacturer's guidelines. Positive and negative controls were included in each diagnostic run.

### Patient management

Patients were contacted with their test result by local clinical staff. Treatment was provided as indicated, in line with Ministry of Health and Medical Services guidelines for the management of STIs, which recommend azithromycin for the treatment of *C. trachomatis* and a third generation cephalosporin for the treatment of *N. gonorrhoeae.*^14^ Contact tracing of partners with a positive test result was conducted in line with national guidelines.

### Sample size

We calculated that a sample size of 200 women was required to have 80% power to show that the prevalence of *C. trachomatis* was ≤10% in the post-MDA sample, as compared with 20% in the pre-MDA sample.^6^ As this was a before-and-after study, we also calculated the sample size required to show a significant difference between women who reported having received and not having received azithromycin during MDA. On the assumption that the *C. trachomatis* prevalence would be unchanged in women who reported not having received MDA (20%) and that it would have declined to 5% in women who were treated during MDA, we calculated that a sample of 75 women who had not received azithromycin during the MDA campaign would be required to show a significant difference between the groups. With a trachoma-programme-estimated MDA coverage of between 75% and 80% we calculated that we would need to recruit approximately 300 women in total.

### Statistical analysis

We report prevalence of STIs stratified by age (16–29 years vs 30–49 years). Exact CIs based on the binomial distribution were calculated for each prevalence estimate. Direct standardisation was used to calculate age-specific prevalence estimates based on the age distribution in the Solomon Islands census.^11^ We used logistic regression to compare the prevalence of STIs both between the pre-MDA and post-MDA surveys after controlling for confounders (age, education, clinic, whether the individual was currently living with a spouse/partner), and between participants in the post-MDA survey who did and did not report receiving treatment during MDA. Logistic regression was also used to estimate unadjusted and adjusted ORs for factors associated with infection, including demographic variables, symptoms of an STI or recent treatment for an STI, and treatment as part of the Ministry of Health trachoma elimination programme. Patients reporting dyspareunia, abnormal vaginal discharge or a genital ulcer in the last month were considered to have symptoms consistent with an STI. All analyses were conducted using Stata V.13.1 (STATACorp, Texas, USA).

## Results

Three hundred and one women were seen at the clinics on study days, of whom 298 (99%) were recruited to this study. Three patients declined to participate. The median age of participants was 25 years (IQR 21–30 years) and the median number of years of education was 9 years (IQR 6–12 years). No women had specifically sought care for current symptoms of an STI but on systematic questioning 23 (7.7%) reported current symptoms consistent with an STI. Twenty-four of 295 women (8.1%) reported treatment for an STI within the previous 12 months. Study participants recruited in the post-MDA survey were slightly younger and slightly more educated than those recruited in the pre-MDA survey (p<0.01 for both comparisons) and slightly more likely to be living with their partner/spouse (p=0.037) (table 1). Only 142/295 women (48.1%) reported having been treated with azithromycin as part of the trachoma campaign. Age and level of education did not differ significantly between women who did and did not report receiving azithromycin MDA (data not shown).

**Table 1:** Demographic characteristics of study participants

	Pre-MDA survey n=296	Post-MDA survey n=298
Age (median and IQR) (years)	28 (23–33)	25 (21–30)
Formal education (median and IQR) (years)	8 (5–11)	9 (6–12)
Ethnicity (%)		
Melanesian	93.8	89.5
Polynesian	3.8	6.8
Micronesian	2.1	2.0
Other	0.4	1
Marital status (%)		
Married	82.4	78.4
Unmarried	17.6	21.6
Currently living with spouse/partner	88.8	87.2

MDA, mass drug administration.

### Diagnostic test results

In the post-MDA survey *C. trachomatis* infection was diagnosed in 43 women (14.4%, 95% CI 10.6% to 18.9%) and *N. gonorrhoeae* in 9 (3%, 95% CI 1.4% to 5.7%). The prevalence of *C. trachomatis* was significantly higher in women aged under 30 years than in those aged 30–49 years (17.7% vs 5.1%, p=0.007) and the prevalence of *N. gonorrhoeae* was also higher in women aged under 30 years than in their older peers, although the latter difference was not statistically significant (3.6% vs 1.3%, p=0.297) (table 2). There was no significant association between a positive diagnostic test for an STI and education level, marital status, living with a partner, current symptoms of an STI or reporting recent treatment for an STI.

**Table 2:** Prevalence of sexually transmitted infections by age group in post-MDA survey

	Overall (n=298)	Age 16–29 years (n=220)	Age 30–49 years (n=78)
*Chlamydia trachomatis*	n=43 14.4% (10.6% to18.9%)	n=39 17.7% (12.9% to 23.4%)	n=4 5.1% (1.4% to 12.6%)
*Neisseria gonorrhoeae*	n=9 3.0% (95% CI 1.4% to 5.7%)	n=8 3.6% (1.6% to 7.0%)	n=1 1.3% (0.3% to 6.9%)

MDA, mass drug administration.

In the combined data set (pre-MDA and post-MDA surveys) only age under 30 years (OR 3.55, 95% CI 1.94 to 6.5, p<0.0001) and not currently living with a partner were associated with a positive test result for *C. trachomatis* (OR 1.74, 95% CI 1.0 to 2.9, p=0.044).

### Impact of MDA on prevalence of bacterial STI

The unadjusted prevalence of *C. trachomatis* had not changed significantly from the pre-MDA survey (14.4% vs 20.3%, p=0.058) (table 3). The age-adjusted prevalence estimate of *C. trachomatis* was 20.7% in the pre-MDA survey and 12.5% in the post-MDA survey (p=0.0073) (table 4). After adjusting for confounders women in the post-MDA survey were significantly less likely to have a positive test result for *C. trachomatis* compared with women in the pre-MDA survey (OR 0.52, 95% CI 0.32 to 0.85, p=0.009). Among study participants in the post-MDA survey, the prevalence of *C. trachomatis* was lower in those who reported receiving treatment during the MDA campaign, but this difference was not statistically significant (11.3% vs 17.8%, p=0.12).

**Table 3:** Prevalence of sexually transmitted infections in the pre-MDA and post-MDA surveys

		Post-MDA survey % (95% CI)		
	Pre-MDA survey (n=296) % (95% CI)	Overall (n=298)*	Reported receiving treatment during MDA (n=142)	Reported not receiving treatment during MDA (n=152)
*Chlamydia trachomatis*	n=60 20.3% (15.9% to 25.4%)	n=43 14.4% (10.6% to 18.9%)	n=16 11.3% (6.6% to 17.7%)	n=27 17.8% (12.0% to 24.8%)
*Neisseria gonorrhoeae*	n=15 5.1% (2.9% to 8.2%)	n=9 3.0% (1.4% to 5.7%)	n=7 4.9% (2.0% to 9.9%)	n=2 1.3% (0.16% to 4.7%)

*Four participants in the post-MDA survey were not sure if they had received MDA or not.

MDA, mass drug administration.

**Table 4:** Age-adjusted prevalence of sexually transmitted infections in the pre-MDA and post-MDA surveys

		Post-MDA survey % (95% CI)		
	Pre-MDA survey	Overall	Reported receiving treatment during MDA	Reported not receiving treatment during MDA
*Chlamydia trachomatis*	20.7% (12.8% to 29.9%)	12.5% (7.7% to 21.4%)	10.6% (4.3% to 25.2%)	14.8% (8.5% to 28.7%)
*Neisseria gonorrhoeae*	5.2% (1.8% to 12.1%)	3.1% (0.8% to 10.0%)	1.1% (1.3% to 18.5%)	5.4% (0.3% to 11.4%)

MDA, mass drug administration.

Neither the unadjusted prevalence of *N. gonorrhoeae* (5.1% vs 3.0%, p=0.20) nor the age-adjusted prevalence (5.3% vs 3.1%, p=0.18) had changed significantly between the pre-MDA and post-MDA surveys, and this remained true after adjusting for confounders (OR 0.57, 95% CI 0.23 to 1.39, p=0.22) (tables 3 and 4). In the post-MDA survey, the difference in the prevalence of *N. gonorrhoeae* between individuals who did and did not report receiving azithromycin during MDA was not statistically significant (4.9% vs 1.3%, p=0.07).

## Discussion

### Key results

In this study we found a 40% decrease in the prevalence of genital *C. trachomatis* infection among female clinic attenders 10 months after azithromycin MDA conducted for the purpose of trachoma elimination. Although the unadjusted decrease in prevalence of *C. trachomatis* was not statistically significant, women enrolled in the post-MDA survey were significantly younger than those in the pre-MDA survey and, after controlling for age, there was strong evidence of a significant decrease in the prevalence of genital *C. trachomatis* infection.

A single round of azithromycin MDA can have a profound and sustained impact on the prevalence of ocular *C. trachomatis* infection.^15^ ^16^ Presumptive mass treatment of sex workers has been shown to reduce the prevalence of STIs among sex workers and their clients in the short term in several studies,^17–19^ but only two published studies have measured its impact in the general population. Studies in Greenland in the 1960s demonstrated a significant impact on gonorrhoea among 15–30 year olds when MDA was combined with clinical examination, laboratory diagnosis and contact tracing but not from MDA alonee.^20^ In the Rakai district of Uganda, five pairs of communities were randomised to receive MDA for either STIs or helminth infection every 10 months.^21^ Treatment coverage was estimated to be between 70% and 80% at each treatment round. At the 20 month follow-up, the prevalence of both syphilis and trichomoniasis was significantly lower in the intervention arm. There was no significant difference between arms in the prevalence of *C. trachomatis* or *N. gonorrhoeae* at 20 months, although the prevalence of *C. trachomatis* fell significantly in the intervention arm from baseline (4.0%) to 20 months (2.4%, p<0.01), having marginally increased in the control arm from 2.2% to 2.6% during the same period.^21^

The significant decrease in *C. trachomatis* prevalence seen in this study 10 months after MDA is encouraging, and suggests that this may be a particularly useful strategy in island populations such as this, where population mobility is likely to be lower than in other settings. The extremely high prevalence of *C. trachomatis* at baseline, compared with most settings, may have increased our ability to detect a significant drop in the prevalence of infection. With data from only one post-MDA time point we cannot exclude the possibility that there was a more profound decrease in the prevalence of genital *C. trachomatis* immediately following MDA, but that this had begun to return to pretreatment levels by the time our impact assessment was conducted. More regular follow-up surveys post MDA should be considered in future studies to better explore the impact of the intervention*.* If a significant effect is confirmed, it will be important to explore the idea that the optimal MDA frequency for control of genital *C. trachomatis* could differ from the annual rounds recommended for trachoma elimination.

### Limitations

Although the impact on the prevalence of genital *C. trachomatis* is encouraging, MDA with azithromycin might potentially induce antimicrobial resistance in other organisms including *N. gonorrhoeae* and *Mycoplasma genitalium.*^22^ Unfortunately laboratory facilities to perform gonococcal culture or testing for *M. genitalium* are not available in the Solomon Islands. Future work, using additional molecular diagnostics not available in the country, is planned using the duplicate swabs collected from study participants during this study, which may allow a preliminary exploration of these questions.

Our study has some weaknesses, the most notable of which is the convenience sampling method. To fully assess the impact of an azithromycin MDA round, a random population sampling design would provide additional useful data. In the Solomon Islands, however, discussion of sexual behaviours remains relatively taboo, and random sampling for a sexual health survey would be unlikely to produce a representative group of study participants. A convenience sample has been used in each of the three previous surveys conducted here. Although the current report describes the ‘after’ component of a before-and-after study, the previous three surveys suggested a steady increase in the prevalence of genital *C. trachomatis* infection among female clinic attenders, from 8% in 2004 to 16% in 2008, rising again to 20% in 2014.^10^ ^23^ Our post-MDA study is the first to show a downward step, which may be taken as further evidence supporting the veracity of the effect we report. Although we cannot exclude a secular trend between our pre-MDA and post-MDA surveys, it seems unlikely that these would account for the significant decrease in the prevalence of genital *C. trachomatis* that we observed.

Second, we lacked an objective measure of treatment uptake during the MDA campaign, so relied on patient report, which is likely to be subject to recall bias.^24^ Unfortunately it was not possible to identify study participants within the routine treatment records produced during MDA. We also lacked information on whether patients’ regular sexual partner(s) had been treated during the MDA campaign, which may partially confound our findings.

### Interpretation

STIs remain a major public health problem in the Pacific, and improved strategies for dealing with this ongoing epidemic are required. The reduction in prevalence of genital *C. trachomatis* infection following MDA for trachoma seen in this study suggests that azithromycin MDA may be one such strategy. Current approaches to syndromic management miss many cases of bacterial STIs.^25^ Other suggested population-based interventions include attempts to increase the coverage of screening for *C. trachomatis* and behavioural interventions to reduce high-risk sexual behaviour, but these are complex and expensive programmes, which have had variable success in reducing the prevalence of STIs.^26^ ^27^ Azithromycin MDA is a well established and safe intervention which has been successfully conducted in a wide range of settings.^13^ ^28^ Prospective evaluations of community mass treatment strategies specifically targeting sexually transmitted *C. trachomatis* should be undertaken.

**Key messages**

- Bacterial sexually transmitted infections (STIs) are common among female clinic attenders in the Solomon Islands.
- There was a 40% reduction in the prevalence of genital Chlamydia infection among this population following a single round of mass treatment with azithromycin.
- Future studies to assess whether community mass treatment may be a viable strategy to control the epidemic of STIs in the Pacific should be performed.
